# NAC1/ACOX2 Axis as a Novel Therapeutic Target for Endometriosis-Related Ovarian Neoplasms

**DOI:** 10.3390/ijms26104938

**Published:** 2025-05-21

**Authors:** Shahataj Begum Sonia, Kentaro Nakayama, Sultana Razia, Naomi Nakayama, Masako Ishikawa, Hitomi Yamashita, Kosuke Kanno, Haruo Takeshita, Umme Farzana Zahan, Hasibul Islam Sohel, Satoru Kyo

**Affiliations:** 1 Department of Obstetrics and Gynecology, Shimane University Faculty of Medicine, Izumo 693-8501, Japan; sbsonia1995@gmail.com (S.B.S.); m-ishi@med.shimane-u.ac.jp (M.I.); meme1103@med.shimane-u.ac.jp (H.Y.); kanno39@med.shimane-u.ac.jp (K.K.); farzanashormi99@gmail.com (U.F.Z.); hasibulsohel1167@gmail.com (H.I.S.); 2 Department of Obstetrics and Gynecology, Nagoya City University East Medical Center, Nagoya 464-8547, Japan; 3 Department of Legal Medicine, Shimane University Faculty of Medicine, 89-1, Enya-Cho, Izumo 693-8501, Japan; raeedahmed@yahoo.com (S.R.); htakeshi@med.shimane-u.ac.jp (H.T.); 4 Department of General Internal Medicine, Nagoya City University East Medical Center, Nagoya 464-8547, Japan; kennaonatsuno@yahoo.co.jp

**Keywords:** NAC1, ACOX2, ovarian cancer, endometriosis, ERON

## Abstract

NAC1, a transcription regulator protein associated with cancer, is highly expressed in several tumor types, including ovarian cancer. However, it remains unclear how NAC1 is involved in carcinogenesis. Our previous studies demonstrated that the knockdown of *NAC1* in ovarian clear cell carcinoma (OCCC) cell lines induces apoptosis and restores their sensitivity to chemotherapy, suggesting NAC1 as a potential therapeutic target. The present study aimed to identify molecular pathways through which NAC1 is involved in the development of endometriosis-related ovarian neoplasms (ERONs). Immunohistochemistry was performed to clarify the relationship between NAC1 and the potential target protein ACOX2 in surgical specimens of ERONs. Reporter assays were conducted to determine the interaction of NAC1 with the specific cis-element on the ACOX2 promoter. Subsequently, a ChIP assay was performed to investigate the in vivo interaction of NAC1 with the ACOX2 promoter. There was an inverse relationship between NAC1 and ACOX2 expressions in the tumor specimens of ERONs. High NAC1/low ACOX2 expression was found to be a worse prognostic marker for patient survival. Reporter assays demonstrated that NAC1 negatively regulated the *ACOX2* promoter via the proximal CATG site. ChIP assays confirmed in vivo binding of NAC1 to the promoter. The present study implicated that NAC1 may contribute to the development of ERONs as a transcriptional repressor by regulating *ACOX2* expression via specific binding sites on the promoter, providing a novel insight into the NAC1/ACOX2 axis as a potential therapeutic target of this tumor type.

## 1. Introduction

Ovarian cancer is widely recognized as the most aggressive gynecological malignancy worldwide, and there has been a remarkable upsurge in its incidence over the past decade [1,2,3]. In the majority of cases, specifically over 70%, tumors have already metastasized beyond the ovaries at the time of diagnosis. The optimal treatment approach for such cases involves a combination of surgical intervention and chemotherapy. Although more than 80% of patients respond to first-line treatments with platinum and taxanes, patients with certain histology types, especially ovarian clear cell cancer (OCCC), are likely to respond poorly.

OCCC, along with ovarian endometrioid carcinoma (OEC), are classified as endometriosis-related ovarian neoplasms (ERONs), as it is now widely accepted that the majority of these tumors develop from benign endometriotic cysts. Since some ERONs that show platinum-resistance represent worse prognosis, especially in advanced or recurrent diseases, drugs targeting specific molecular pathways for the development of ERONs are needed [4,5,6,7,8,9], and a clearer understanding of the molecular carcinogenesis of ERONs is a prerequisite to designing such specific chemotherapeutic agents [10,11].

Genes of the BTB/POZ (broad complex, tramtrack, bric-à-brac/poxvirus, and zinc finger) (hereafter abbreviated BTB) family have been known to participate in various cellular functions, including transcription regulation, cellular proliferation, apoptosis, and cell morphology maintenance [12]. Recently, nucleus accumbens-associated protein 1 (NAC1), encoded by the *NACC1* gene, was identified as a carcinoma-associated BTB/POZ family member [13]. NAC1 is significantly overexpressed in several carcinoma types, including ovarian, colorectal, breast, renal cell, cervical, and pancreatic carcinomas, and is associated with tumor growth and survival via resistance to platinum-based chemotherapy [13,14,15,16,17,18,19,20,21,22,23]. The levels of NAC1 expression correlate with tumor recurrence in ovarian serous carcinomas, and intense NAC1 immunoreactivity in primary ovarian tumors predicts early recurrence [21]. Additionally, the expression of NAC1 correlates with taxane resistance in advanced-stage ovarian cancer [13,24]. These reports suggested that NAC1 plays various functional roles in ovarian cancer development and that it might be a potential therapeutic target.

NAC1 encompasses the N-terminal BTB/POZ and C-terminal BEN (BANP, E5R, and NAC1) domains. The BTB domain, spanning approximately 100 amino acids, is a conserved motif known for mediating both homodimerization and/or heterodimerization and facilitating interactions with other proteins [17,25]. NAC1 homodimerizes via its BTB domain [12] and heterodimerizes with Myc-interacting zinc finger protein 1 (Miz1) via the respective BTB domain [26,27]. Computational analysis has identified the BEN domain in mediating protein–DNA and protein–protein interactions [28], and a recent study has demonstrated that NAC1 functions as a sequence-specific DNA-binding domain, directly interacting with DNA through the BEN domain [29].

To determine the molecular mechanisms underlying how NAC1 expression contributes to the growth and survival of tumor cells overexpressing NAC1, we previously used microarray profiling and sought to identify differentially expressed genes in cells with and without the knockdown of *NAC1* [Unpublished data]. We identified and validated several genes whose expression was up-regulated by *NAC1* knockdown and thus negatively regulated by NAC1 molecules. One of the genes negatively regulated by NAC1 was acyl-CoA oxidase 2 (ACOX2), a member of the ACOX protein family located on chromosome 3p14.319 [30]. ACOX2 is primarily associated with fatty acid metabolism, particularly in the oxidation of very long-chain fatty acids (VLCFAs) and branched-chain fatty acids (BCFAs). This process generates acetyl-CoA units for energy production and contributes to membrane lipid metabolism and signaling molecule synthesis. The dysregulation of ACOX2 may impact cellular metabolism and is implicated in various diseases, including ovarian cancer [31,32,33,34,35,36,37]. Analysis of the TCGA dataset revealed elevated ACOX2 expression compared to other gynecological cancer types [38].

This background prompted us to investigate the underlying regulatory mechanism by which NAC1 modulates the expression of ACOX2, ultimately elucidating its role in the development of ERONs

## 2. Results

### 2.1. NAC1 Expression Is Inversely Associated with ACOX2 Expression in ERONs

**Figure 1:**
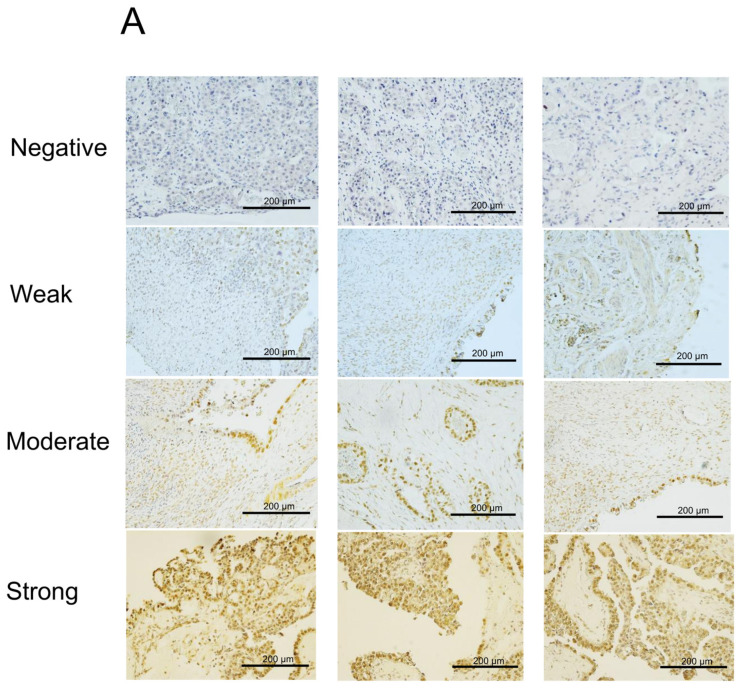

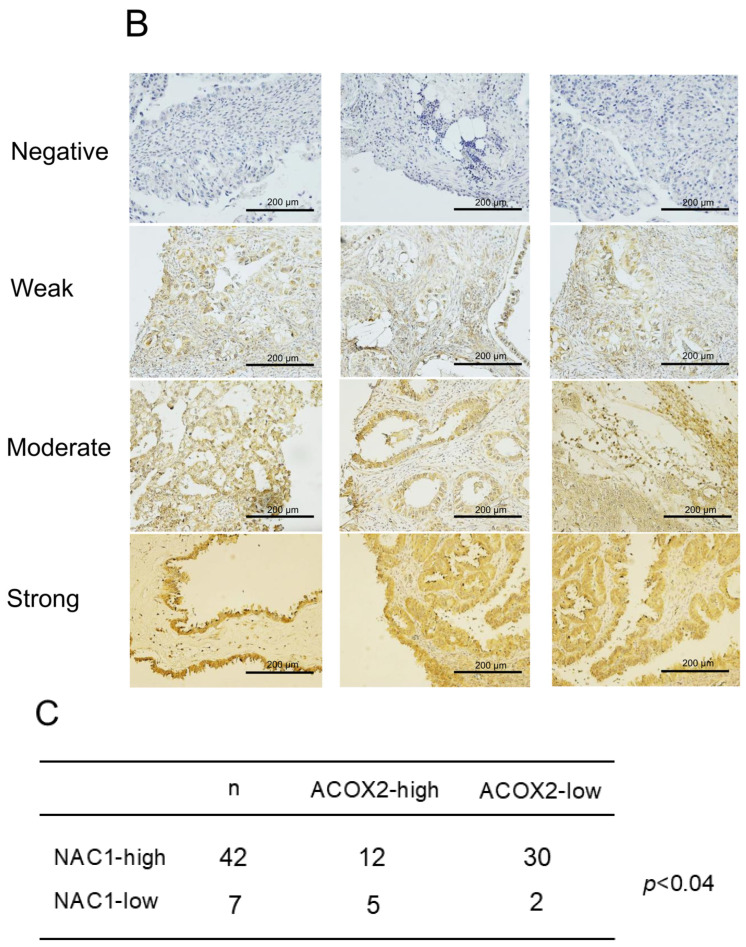
Immunohistochemistry of NAC1 and ACOX2 in ERON surgical specimens. Representative cases showing negative (0), weak (1+), moderate (2+), and strong (3+) staining intensities are presented for NAC1 (**A**) and ACOX2 (**B**). Images were taken from three different plots for each expression category. The correlation between NAC1 and ACOX2 expression was evaluated via Fisher’s exact test (**C**).

We first examined the expression of NAC1 in ERON surgical specimens using immunohistochemistry. Nuclear staining of NAC1 was observed in tumor cells, and 42 of the 49 cases examined (86%) were classified as high NAC1 expression (Figure 1A). In contrast, ACOX2 staining was constrained to the cytoplasm (Figure 1B), as previously described [33]; 17 cases showed high ACOX2 expression and 32 exhibited low expression. Among the 42 cases with high NAC1 expression, 12 showed high ACOX2 expression (29%) and 30 exhibited low expression (71%). Among the seven cases with low NAC1 expression, five showed high ACOX2 (71%) expression and two had low (28%) expression. Therefore, expression levels between the two proteins showed a negative correlation (*p* < 0.04).

### 2.2. Increased NAC1 and Decreased ACOX2 Expressions Are Associated with Worse Prognosis in Patients with ERONs

**Figure 2:**
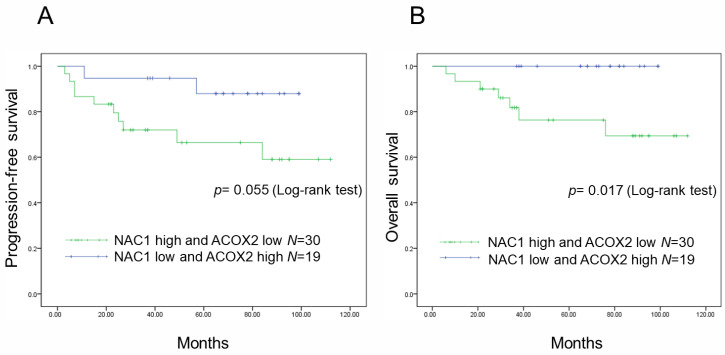
Prognostic significance of NAC1 and ACOX2 expressions in patients with ERONs. Kaplan–Meier survival analyses are shown for high NAC1/low ACOX2 expression in relation to progression-free survival (PFS) (**A**) and overall survival (OS) (**B**). M: months.

Patients with ERONs were divided into two groups based on the expression status of NAC1 and ACOX2, and PFS and OS were analyzed according to the expression status. Patients with high NAC1 expression had significantly shorter PFS and OS (*p* = 0.272, *p* = 0.057, respectively; log-rank test). In contrast, patients with low ACOX2 expression showed significantly shorter PFS and OS (*p* = 0.002, *p* = 0.011, respectively; log-rank test) (Supplementary Figure S1). When both expressions were combined, PFS was likely to be worse in patients with high NAC1 and low ACOX2 expressions compared with those with low NAC1 and high ACOX2 expressions (*p* = 0.055; log-rank test) (Figure 2A). Additionally, patients with high NAC1 and low ACOX2 expressions showed statistically significant shorter OS compared to those with low NAC1 and high ACOX2 expressions (*p* = 0.017; log-rank test) (Figure 2B). These results suggest that the combined expression status of high NAC1/low ACOX2 can serve as a prognostic indicator in patients with ERONs.

### 2.3. NAC1 Negatively Regulates ACOX2 Expression in OCCC Cells

**Figure 3:**
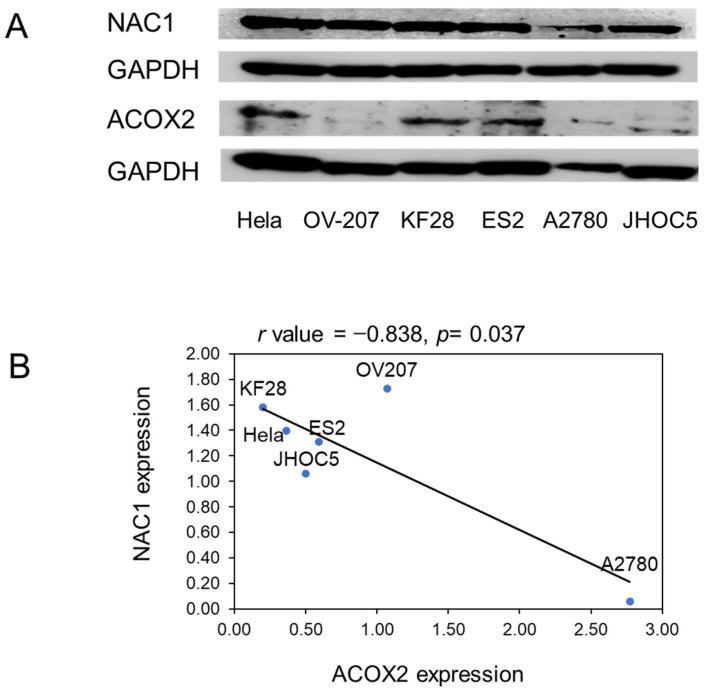
Pearson’s correlation between NAC1 and ACOX2 expression in OCCC cells. Representative results of Western blot analysis of NAC1 and ACOX2 in each OCCC cell line (**A**) and their correlation by quantitative evaluation (**B**) measured by ImageJ software (version 1.53k). The full-length Western blot gel image is available in Supplementary Figure S2.

**Figure 4:**
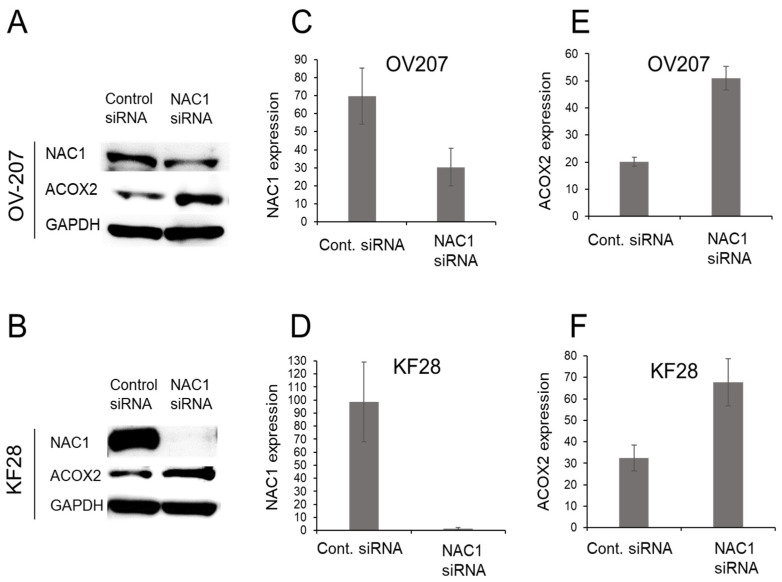
siRNA-knockdown analysis of NAC1 in OCCC cell lines. Western blotting was used to evaluate NAC1 and ACOX2 protein expressions in OV207 and KF28 OCCC cell lines after siRNA knockdown (**A**,**B**). *NAC1* knockdown was confirmed in both cell lines (**C**,**D**), and efficient knockdown of NAC1 led to significant upregulation of ACOX2 in both cell lines (**E**,**F**). The full-length Western blot gel image is available in Supplementary Figure S3.

The levels of NAC1 and ACOX2 expressions were evaluated in several OCCC cell lines. Western blot analyses showed a significantly negative correlation between both protein expressions (Figure 3), as observed in clinical specimens of ERONs. To investigate the potential regulatory effect of NAC1 on ACOX2 expression, an siRNA knockdown assay was performed using two types of OCCC cell lines, OV207 and KF28 (Figure 4A,B). We found that the efficient knockdown of *NAC1* resulted in significantly increased ACOX2 expression in both cell lines (Figure 4C,D). These findings underscore that NAC1 negatively regulates ACOX2 expression in OCCC cells.

### 2.4. NAC1 Represses ACOX2 Promoter Transcriptional Activity in OCCC Cell Lines

**Figure 5:**
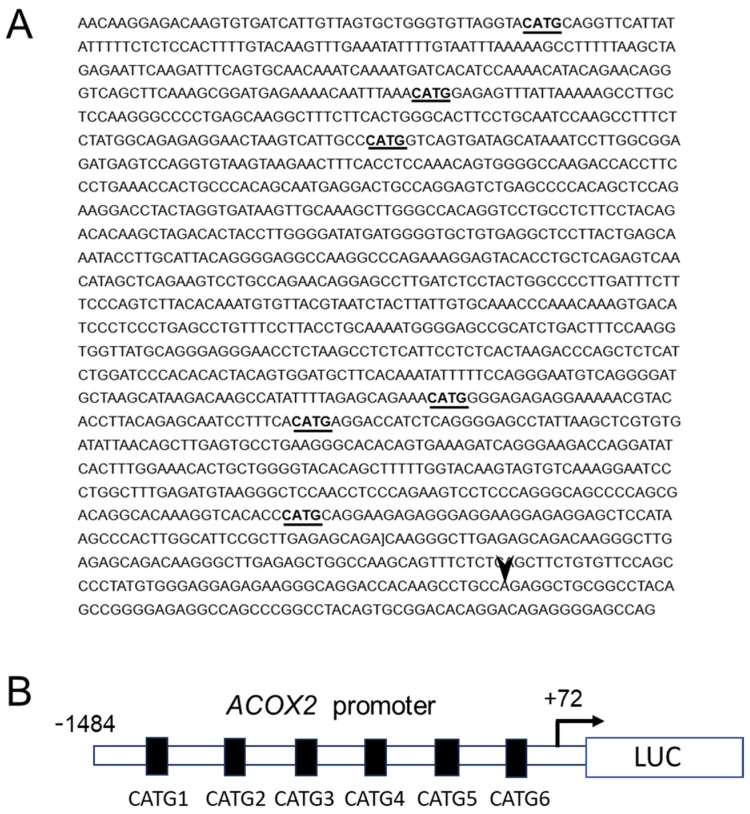

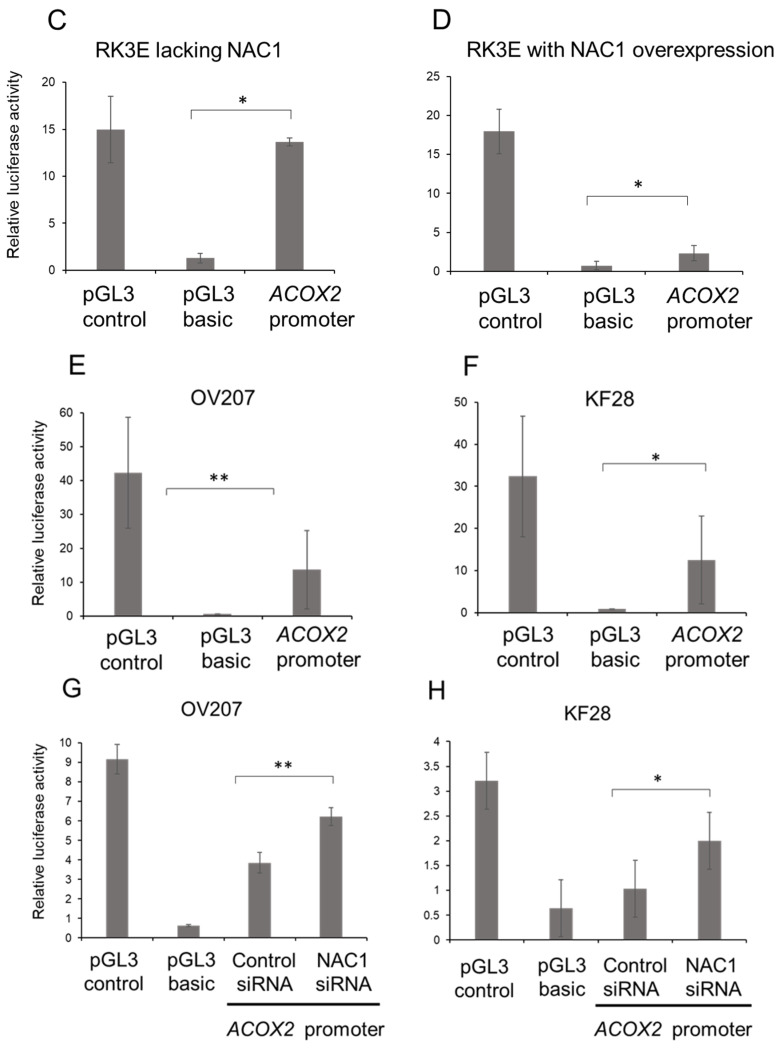
Transcriptional activity of the *ACOX2* promoter in OCCC cell lines with different levels of NAC1 expression. The promoter sequence of the ACOX2 gene spans from −1488 to +72 upstream of the transcriptional start site (arrowhead) (**A**). The underlines indicate the 6 CATG consensus motifs for NAC1 binding. (**B**) The luciferase (LUC) reporter construct, in which the *ACOX2* full-length promoter was inserted into the pGL3 basic vector, was prepared (**B**) and applied to the luciferase reporter assays. The transcriptional activity of the *ACOX2* promoter was evaluated in each reporter construct using RK3E cells lacking endogenous expression of NAC1 (**C**) or those with NAC1 overexpression (**D**). The promoter activity of *ACOX2* was further evaluated in OV207 and KF28 cells, both expressing endogenous NAC1 expression (**E**,**F**). NAC1 in each cell type was then knocked down by siRNA, and the promoter activity of *ACOX2* was also evaluated (**G**,**H**). Each experiment was independently repeated three times with three technical replicates. Statistical significance is represented as * *p* < 0.05; ** *p* < 0.005.

We next sought to investigate whether NAC1 regulates ACOX2 expression at the *ACOX* transcription level. We constructed luciferase reporter plasmids, in which the promoter sequences spanning from +72 to −1488 upstream of the transcriptional start sites of the human *ACOX2* gene (Figure 5A,B) were inserted into a pGL3 basic vector. The luciferase reporter assays were then performed using OCCC cells with or without NAC1 expression.

RK3E cells lack endogenous NAC1 expression. In this cell type, the transcriptional activity of the *ACOX2* promoter showed significantly higher levels compared to the basic promoter activity (Figure 5C). Then, we overexpressed NAC1 in RK3E cells by transfecting the expression vector of *NAC1*, followed by the luciferase assay. The promoter activity of *ACOX2* was found to be maintained at relatively low levels (Figure 5D).

We next examined the transcriptional activity of *ACOX2* in OV207 and KF28 cell lines with endogenous NAC1 expression. The transcriptional activity of *ACOX2* was maintained at relatively low levels in both cell lines compared to positive control promoters (Figure 5E,F). However, when *NAC1* was knocked down in both cell lines, the transcriptional activity was significantly increased compared to those treated with control siRNA. Overall, these data suggest that human *ACOX2* gene promoter activity is negatively regulated by NAC1 (Figure 5G,H), confirming that NAC repressed *ACOX* expression at the level of transcription.

### 2.5. The Proximal CATG Site on the ACOX2 Promoter May Be Responsible for the Transcriptional Repression by NAC1

**Figure 6:**
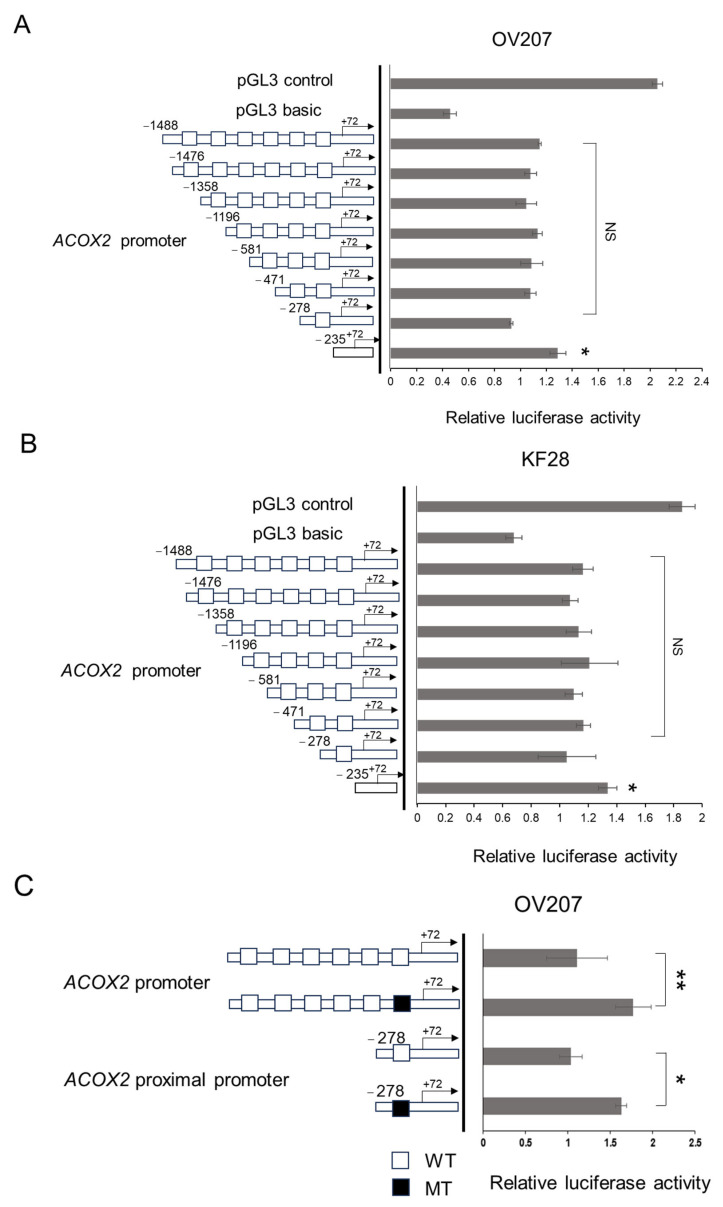

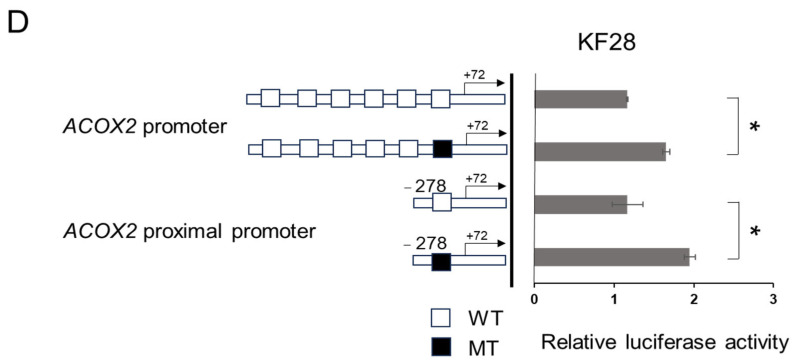
Promoter assays to identify cis-elements of the ACOX2 promoter responsible for NAC1 repression. (**A**,**B**) The full-length or 5′ deleted promoter sequences containing different numbers of CATG sites were prepared and incorporated into the luciferase reporter constructs, followed by luciferase assays using OV207 and KF28 cells. Furthermore, mutant reporter plasmids, in which the most proximal CATG sites were substitution-mutated into the full-length promoter or 5′ deleted promoter containing only this site, were prepared (**C**,**D**), followed by luciferase assays using both cell lines. WT: wild type CATG site. MT: substitution mutated CATG site. Each experiment was independently repeated three times with three technical replicates. Statistical significance is represented as * *p* < 0.05; ** *p* < 0.005. NS: Not significant.

We previously found six consensus motifs, CATG, for potential NAC1 binding [30] in the *ACOX2* promoter via a genome browser search, which were designated as CATG 1 (−1476 to +72), CATG 2 (−1358 to +72), CATG 3 (−1196 to +72), CATG 4 (−581 to +72), CATG 5 (−471 to +72), and CATG 6 (−278 to +72) (Figure 5A). To analyze the interaction of NAC1 with each site, we generated a series of deletion fragments of the *ACOX2* promoter in which distal promoter sequences were sequentially deleted (Figure 6A,B). The full-length or deleted promoter fragments were incorporated into luciferase reporter plasmids, transfected into OV207 and KF28 cells, and luciferase assays were performed. Unexpectedly, the transcriptional activity of each *ACOX2* promoter fragment was not significantly altered, similar to that of the full-length promoter in both cell lines. However, the promoter fragment lacking any of the CATG sites (−235 to +72) showed significantly increased transcriptional activity. These data suggest that there must be a complex regulatory interplay involving NAC1 to modulate the *ACOX2* promoter and that CATG sites may not largely be involved in transcriptional repression, while the most proximal CATG site (CATG 6) can at least partly be responsible for NAC1 action as a cis-element.

To further confirm the contribution of the most proximal CATG 6 site to NAC1 repression, we constructed mutant reporter plasmids in which this site was substitution-mutated from CATG to AAAA in full-length or proximal (−278 to +72) promoters, followed by the luciferase assay using OV207 and KF28 cells (Figure 6C,D). We found that the introduction of this mutation led to an increase in the full-length or proximal promoter activity in both cell lines. Thus, the most proximal CATG 6 site may be responsible for NAC1 inhibition of the *ACOX2* promoter.

### 2.6. In Vivo Binding of NAC1 to the Most Proximal CATG Site on the ACOX2 Promoter

**Figure 7:**
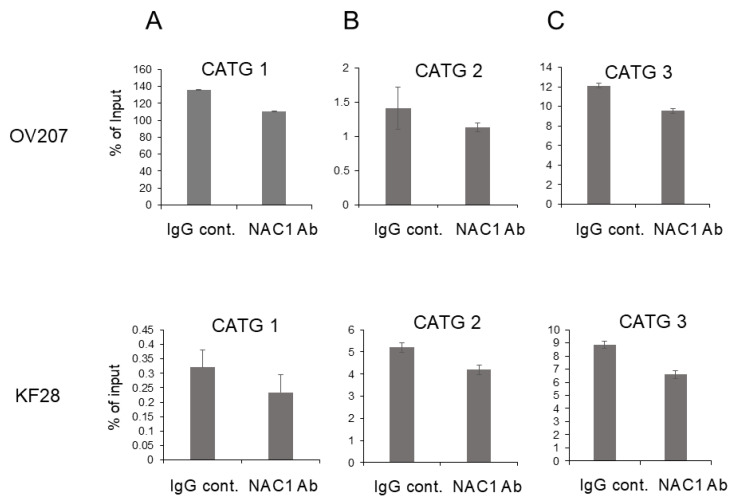

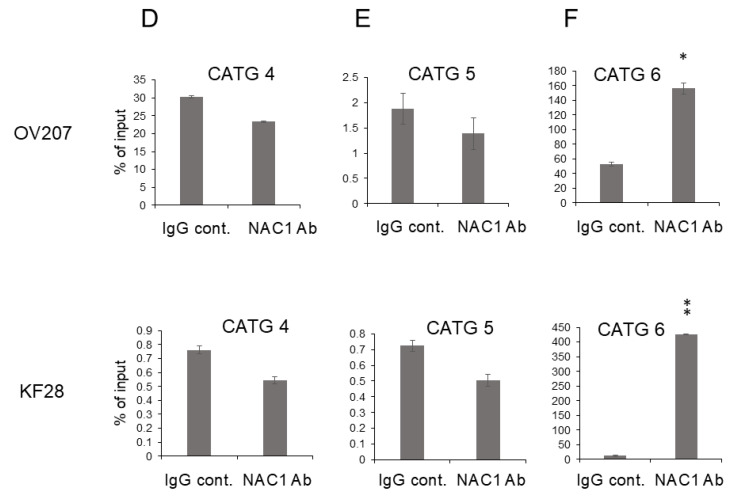
ChIP analysis to identify the binding of NAC1 to each CATG sequence on the *ACOX2* promoter using OV207 and KF28 cells. Primer sets for quantitative PCRs targeting each CATG 1, 2, 3, 4, 5 site generated equivalent levels of immunoprecipitates by the NAC1 antibody compared to those with the control IgG in both cell lines (**A**–**E**). In contrast, primer sets targeting the most proximal CATG 6 site generated apparently increased levels of immunoprecipitates compared to those with control IgG in both cell lines (**F**). Normal rabbit IgG (IgG) served as a control antibody, while NAC1 antibody (Ab) was used to detect NAC1 binding. The data are shown as mean values relative to the input, expressed as a percentage (% of input). The error bars indicate the standard deviation, based on three independent measurements (*n* = 3). Statistical significance is represented as * *p* < 0.05; ** *p* < 0.005.

We finally performed a ChIP assay to examine in vivo binding of NAC1 to the *ACOX2* promoter. Chromatin fragments isolated from OV207 and KF28 cells were immunoprecipitated with an anti-NAC1 antibody. The immunoprecipitates were then examined for the presence of each of the CATG sites (CATG 1, CATG 2, CATG 3, CATG 4, CATG 5, and CATG 6) by PCR amplification using site-specific primers. We found that the amounts of PCR products from the immunoprecipitates with NAC1 antibody were not significantly altered compared to those with control IgG antibody using primer sets for CATG1-5 sites (Figure 7A–E). However, the primer sets spanning the most proximal CATG 6 site successfully amplified PCR products more efficiently with the NAC1 antibody compared to the control antibody (Figure 7F). These findings indicate that the most proximal CATG 6 sequence may be the potential NAC1 binding site on the *ACOX2* promoter.

### 2.7. NAC1 Knockdown Suppresses Fatty Acid Metabolism Genes in OCCC Cell Lines

**Figure 8:**
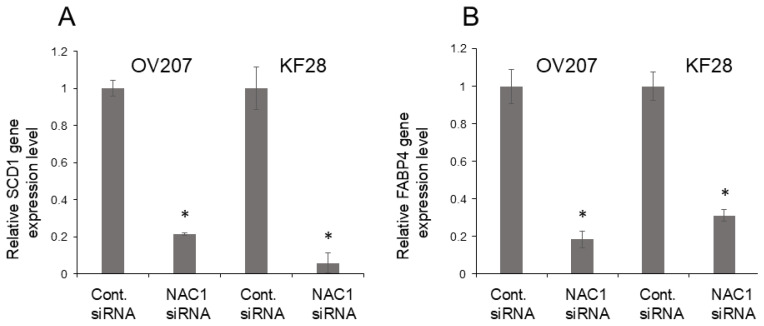
Association between NAC1 expression and key fatty acid metabolism-related genes. Gene expression analysis demonstrates a significant reduction in stearoyl-coenzyme A desaturase 1 (SCD1) and fatty acid-binding protein 4 (FABP4) expression in NAC1 siRNA-treated cells compared to control siRNA-treated cells in OV207 and KF28 cell lines (**A**,**B**). The error bars indicate the standard deviation, based on three independent measurements (*n* = 3). Statistical significance is represented as * *p* < 0.05.

After performing gene expression analysis, we found that the reduction of *NACC1* gene expression significantly inhibited the expression of SCD1 and FABP4 in OCCC cell lines OV207 and KF28 (Figure 8A,B). These results suggest that NAC1 may play a potential role in the regulation of fatty acid metabolism.

## 3. Discussion

In the present study, we demonstrated the upregulation of NAC1 in clinical specimens of ERONs and the inverse relationship between NAC1 and ACOX2 expression, which was further confirmed by in vitro experiments using OCCC cells. We next sought to clarify the molecular mechanisms of this inverse relationship. Reporter and ChIP assays identified the biological interaction between NAC1 and ACOX2, and NAC1 plays a vital role as a transcription factor that represses the transcriptional activity of *ACOX2* via binding to a specific CATG site on its promoter. The present groundbreaking discovery marks the first evidence of NAC1’s involvement in the downregulation of *ACOX2* in ERONs.

Recent studies indicate that NAC1 functions as a transcriptional repressor in various types of cancer, interacting with other proteins via its POZ/BTB domain, which forms complexes capable of suppressing the transcription of target genes. This repressive role has been consistently observed across diverse cancers, including ovarian and endometrial cancers [14,18,27,39]. Notably, NAC1 has been found to suppress the expression of genes associated with tumor suppression and differentiation, thereby fostering tumor cell proliferation and survival [14,18,39,40,41], consistent with the present finding that NAC1 suppresses ACOX2 expression to contribute to the development of ERONs.

**Figure 9:**
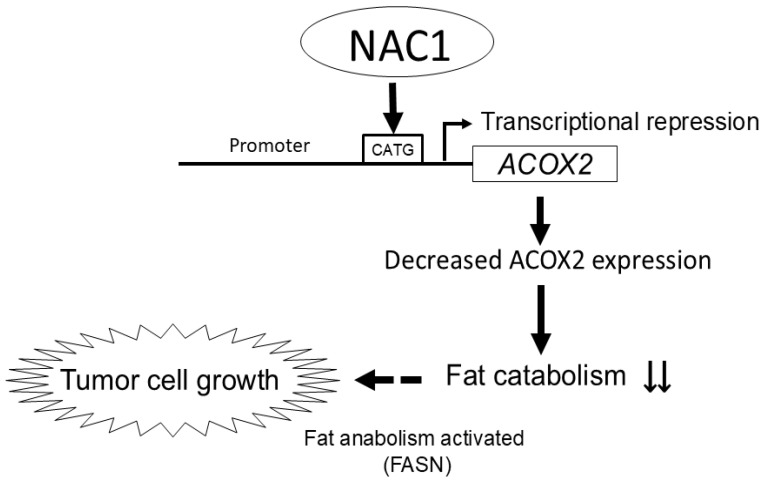
Proposed model for the role of NAC1 in the development of ERONs via the regulation of ACOX2 expression.

Previous research has demonstrated that reduced ACOX2 activity can lead to a shift towards anabolic processes, providing cancer cells with the necessary building blocks for rapid proliferation and growth [42]. When cancer cells experience metabolic stress, NAC1 is overexpressed, which may enhance its binding to the *ACOX2* promoter to repress ACOX2 expression, leading to decreased fat catabolism and increased fat anabolism. Consequently, this shift can promote tumor cell growth (Figure 9).

To address the concerns regarding the functional impact of NAC1 on fatty acid metabolism, we investigated the expression of key lipid metabolism-related genes. A recent report identified a total of 38 genes involved in lipid metabolism during cancer progression. Among these, stearoyl-coenzyme A desaturase 1 (SCD1) was the most frequently reported, followed by fatty acid synthase (FASN) and fatty acid-binding protein 4 (FABP4) [43,44,45,46,47,48]. In our previous work, we demonstrated that NAC1/FASN overexpression is critical for the growth and survival of a subset of ovarian clear cell carcinomas (OCCC). NAC1 overexpression enhanced FASN expression, while NAC1 silencing via siRNA reduced FASN levels in OCCC cell lines [49,50]. In our current study, we observed that NAC1 knockdown also led to a decreased expression of SCD1 and FABP4. These results suggest that NAC1 may play a significant role in regulating fatty acid metabolism, as its silencing reduces the expression of key genes involved in fatty acid synthesis and processing. Although these findings are based on gene expression analyses, we plan to conduct metabolic assays in future studies to directly evaluate changes in fat metabolism at the functional level.

A limitation of the present study is a lack of in vitro and/or in vivo analyses to verify the oncologic effects of NAC1 and ACOX2, possibly by overexpressing or knocking down each gene in OCCC cells. Furthermore, we did not observe the prognostic impact of NAC1/ACOX2 in other histological subtypes of ovarian cancer in order to clarify whether our findings are ERON-specific. Lastly, while we could clearly confirm the in vivo binding of NAC1 to the *ACOX2* promoter, it remains unclear whether this binding is a direct interaction or an indirect binding via complex formation with other co-factors.

A strength of the study is that we dissected the precise molecular mechanisms of ACOX2 repression by NAC1, in which the proximal CATG on the ACOX2 promoter was essential as a cis-element, for the first time. In addition, to our knowledge, this study is the first report to prove the prognostic impact of the NAC1/ACOX2 axis; high levels of NAC1 combined with low levels of ACOX2 were associated with worse prognosis in patients with ERONs, signifying a novel prognostic marker for this tumor type. Similar observations were made in other tumor types, including hepatocellular carcinoma and breast cancer, albeit expressing either NAC1 or ACOX2, demonstrating that the overexpression of NAC1 or the low expression of ACOX2 was correlated with shorter disease-free survival in these tumors [20,33,34,35,51], thus both factors might be mutually correlated to affect oncologic outcomes across tumor types.

In summary, the present study establishes a concept in which NAC1 plays a role in the development of ERONs via controlling ACOX2 expression through specific binding sites on its promoter. This highlights that the NAC1/ACOX2 axis is a potential molecular target as both a prognostic indicator and a novel therapeutic strategy.

## 4. Materials and Methods

### 4.1. Cell Lines

The human ovarian clear cell carcinoma cell lines OV207, KF28, and RK3E were obtained from the American Tissue Culture Center (Rockville, MD, USA). Cell line characterization and authentication were performed using morphology, karyotyping, PCR, and STR profiling by the cell bank, ATCC. To ensure the integrity of the cell lines, multiple aliquots of frozen stocks were prepared from the initial stocks, and every 3 months, new frozen stock was utilized for the experiments. The cells were routinely inspected for identity confirmation by morphology and growth curve analysis and validated to be mycoplasma-free.

### 4.2. Tissue Samples

Formalin-fixed, paraffin-embedded (FFPE) surgical samples of 49 ERONs, consisting of 36 cases of OCCC and 13 cases of OEC, were obtained from patients at the Department of Obstetrics and Gynecology at Shimane University Hospital. Pathological diagnosis was based on conventional morphological examination of hematoxylin and eosin-stained sections, which were classified according to the WHO classification. Tumor staging was performed according to the International Federation of Gynecology and Obstetrics (FIGO) classification. The acquisition of tissue specimens and clinical information was approved by an institutional review board (Shimane University) (approval number 2004-0381, 5 March 2007).

### 4.3. Immunohistochemistry

Immunohistochemistry analysis was performed using FFPE tissue sections. FFPE sections (4 µm thick) were dewaxed in xylene and hydrated using graded alcohol. Antigen retrieval was performed by autoclaving at 121 °C using TE buffer, and subsequently, slides were incubated for 30 min in phosphate-buffered saline (PBS) with 3% H_2_O_2_ and rinsed thrice with PBS. The slides were then incubated overnight at 4 °C with antibodies against a monoclonal anti-NAC1 (9.27) antibody [52] or polyclonal anti-ACOX2 (Atlas Antibodies, Stockholm, Sweden) at a dilution of 1:200. The HRP-conjugated secondary antibodies were added to the sections on the slides and incubated in a humidified chamber at room temperature for 30 min before visualization with DAB substrate solution. The slides were then dehydrated with graded alcohol, cleared with xylene, and coverslips were mounted using mounting solution. The color of the antibody staining in the tissue sections was observed under light microscopy. For the assessment of NAC1 and ACOX2 immunoreactivity, the H-score method was applied to evaluate the staining score of each sample, which was calculated by using the percentage of positive-stained carcinoma cells (from 0 to 100) and the staining intensity (graded as 0, non-staining; 1, weak; 2, moderate; and 3, strong). The range of the H-score was from 0 to 300. Based on the H-score, the stained samples were divided into four groups: 0, 1+, 2+, and 3+. The levels 0, 1+ were considered as low, and 2+ and 3+ were considered as high [51,53].

### 4.4. Western Blotting

Cell pellets were lysed in lysis buffer, heated for 5 min at 100 °C, and then placed on ice for 1 min. LDS buffer and sample-reducing buffer were added, and samples were centrifuged for 5 min at 15,000 rpm. Duplicate samples with a protein concentration of 10 µg were subjected to SDS-polyacrylamide gel electrophoresis and transferred to polyvinylidene fluoride membranes. Membranes were blocked in 5% skim milk with TBST for 1 h, probed with NAC1 antibody (dilution, 1:250; Abcam, Cambridge, MA, USA) or ACOX2 antibody (dilution, 1:700; Sigma-Aldrich, St. Louis, MO, USA)), and incubated overnight at 4 °C on a shaker. Membranes were washed four times with TBST and incubated with either anti-rabbit IgG-HRP (sc-2357) or anti-mouse (1:10,000) peroxidase-conjugated secondary antibodies for 1 h at room temperature. As a loading control, membranes were probed with an antibody targeting GAPDH (dilution, 1:10,000) from Cell Signaling Technology (Beverly, MA, USA). Protein bands were visualized using an Amersham ImageQuant 800 scanner (Cytiva, Marlborough, MA, USA) and raw intensity and near-infrared fluorescence values were determined using ImageQuant 8.1 analytical software. Intra-lane background signals were removed, and boxes were manually adjusted over each desired band.

### 4.5. Targeted Gene Silencing RNA Knockdown of NAC1 Gene Expression

Stealth small interfering RNA (siRNA) against NAC1 (#1, 5′-CCGGCUGAACUUAUCAACCAGAUUG-3′) [54] was purchased from Thermo Fisher Scientific (Waltham, MA, USA). Cells were seeded onto 6-well plates and transfected with siRNAs using RNAiMAX (Thermo Fisher Scientific) according to the manufacturer’s instructions. The initial cell seeding density was (8 × 10^5^ cells) per well. After a 48-h incubation period, luminescence was measured using a luminometer. The data are presented as the means ± 1 standard deviation of triplicate determinations.

### 4.6. Luciferase Assay Assessment of Functional Activity

The full-length promoter of human *ACOX2*, along with the deletion mutants with or without substitution mutation (from CATG to AAAA) within the most proximal CATG site, was prepared by PCR-based mutagenesis using site-specific primers and incorporated into the luciferase reporter pGL3-basic (Promega, Madison, WI, USA). The pGL3-control vector was used as a positive control of the luciferase assay (Promega).

The luciferase assay was performed using OCCC cell lines OV207, KF28, and RK3E according to the manufacturer’s protocol. Luciferase activity of the supernatant was measured with the Dual-Luciferase Reporter Assay System (Promega) using a luminometer (TD-20/20) (Promega). Each experiment was independently repeated three times with three technical replicates.

### 4.7. Chromatin Immunoprecipitation (ChIP) Assay

To generate mouse monoclonal antibodies, the region encompassing amino acids 284–357 of human NAC1 was expressed as His-tagged proteins in *Escherichia coli*. Human ovarian OV207 and KF28 cells were cross-linked with a final concentration of 1% (*v*/*v*) formaldehyde for 10 min at 37 °C; then, the cells were incubated for 5 min at 37 °C in 0.125 M glycine to stop the reaction. After washing with ice-cold PBS and RIPA lysis buffer, the cells were lysed with 600 µL of nuclear lysis buffer (1% SDS, 10 mM EDTA, 50 mM Tris, pH 8.1) and protease inhibitors for 10 min on ice. Then, a Branson sonifier 250 (Brookfield, CT, USA) was used to shear the genomic DNA. After the removal of cellular debris by centrifugation and digestion of the RNA with RNase A, equal amounts of DNA were incubated with 2 µg of anti-NAC1 antibody or control IgG antibodies previously bound to anti-rabbit IgG-coupled magnetic beads (Dynabeads M-280; Thermo Fisher Scientific, Waltham, MA, USA). After extensive washing, the precipitated DNA fragments were eluted. Precipitated DNA was analyzed by quantitative real-time PCR using the primer sets covering each CATG site promoter sequence as shown below.
Sequence name (5′- to -3′)
CATG 1 FAAGGAGACAAGTGTGATCATTGTTACATG 1 RGCTTTGAAGCTGACCCTGTTCATG 2 FGCCTTTTTAAGCTAGAGAATTCAAGACATG 2 RGAGAAAGGCTTGGATTGCAGCATG 3 FTTCTTCACTGGGCACTTCCTCATG 3 RTTTCAGGGAAGGTGGTCTTGCATG 4 FTCTCATCTGGATCCCACACACATG 4 RTGAAAGGATTGCTCTGTAAGGTGCATG 5 FGGGAGAGAGGAAAAACGTACACATG 5 RCCCAGCAGTGTTTCCAAAGTCATG 6 FGAATCCCTGGCTTTGAGATGCATG 6 RACATAGGGGCTGGAACACAGF: forward, R: reverse.


Chip-qPCR experiments were performed in at least triplicate on independent biological samples.

### 4.8. Quantitative Real-Time RT-PCR (qRT-PCR)

The siRNA preparation and RNA interference were performed over 72 h using lipofectamine^®^ RNAiMAX (Invitrogen, Carslsbad, CA, USA), according to the manufacturer’s protocol. RNA was extracted using the RNeasy Mini Kit (QIAGEN, Venlo, The Netherlands) according to the manufacturer’s instructions, and concentrations were determined using a NanoDrop ND-1000 spectrophotometer (Thermo Fisher Scientific, Waltham, MA, USA). First-strand cDNA synthesis and amplification were performed using reverse transcription reagents (Thermo Fisher Scientific, Inc., Waltham, MA, USA), according to the manufacturer’s guidelines. RT–PCR was performed using the TOYOBO RT-PCR kit (TOYOBO, Osaka, Japan). Primers used for amplification and PCR cycles were as follows: human SCD1 (SCD) forward primer sequence 5′-CCTGGTTTCACTTGGAGCTGTG-3′ and reverse primer 5′-TGTGGTGAAGTTGATGTGCCAGC-3′; human FABP4 forward primer sequence 5′-ACGAGAGGATGATAAACTGGTGG-3′ and reverse primer 5′-GCGAACTTCAGTCCAGGTCAAC-3′; GAPDH forward primer sequence 5′-TGTTGCCATCAATGACCCCTT-3′ and reverse primer 5′-CTCCACGACGTACTCAGCG-3′. The standard PCR conditions were set as follows: 95 °C for 30 s, 95 °C for 5 s, and 60 °C for 30 s; initial denaturation followed by 40 cycles of amplification at 95 °C for 15 s, 60 °C for 30 s, and 95 °C for 15 s. The data were normalized to GAPDH levels and expressed as relative mRNA levels. The experiment was repeated independently at least three times.

### 4.9. Statistical Analysis

Progression-free survival (PFS) and overall survival (OS) were calculated from the date of diagnosis to the date of first relapse or last follow-up, standardizing the methodology. Kaplan–Meier curves were plotted to represent the data, and the significance of differences was assessed using Student’s *t*-test with SPSS software (version 21; IBM, Armonk, NY, USA). A *p*-value < 0.05 was deemed statistically significant. Fischer’s exact test was employed for comparisons involving categorical data. Protein expression levels were determined by measuring the band intensities on Western blots using Image J software (version 1.53k). The Pearson correlation coefficient was calculated to assess the linear relationship between variables using SPSS software (version 21; IBM, Armonk, NY, USA). A *p*-value < 0.05 was considered statistically significant.

## Data Availability

The data presented in this study are available on request from the corresponding author (S.K. and K.N.).

## Supplementary Materials

The following supporting information can be downloaded at https://www.mdpi.com/article/10.3390/ijms26104938/s1.

## Abbreviations

The following abbreviations are used in this manuscript:

NAC1	Nucleus accumbens-associated protein 1
ACOX2	Acyl-CoA oxidase 2
OCCC	Ovarian clear cell carcinoma
ERON	Endometriosis-related ovarian neoplasms
SCD1	Stearoyl-coenzyme A desaturase 1
FABP4	Fatty acid-binding protein 4
