# Supplementary material for: NAC1/ACOX2 Axis as a Novel Therapeutic Target for Endometriosis-Related Ovarian Neoplasms
Source: Int J Mol Sci. 2025 May 21;26(10):4938. doi: 10.3390/ijms26104938 (PMC12112610; doi:10.3390/ijms26104938)
Supplement: Supplementary file 1 [file ijms-26-04938-s001.zip › ijms-3548019-supplementary.pdf]

Article

NAC1/ACOX2 axis as a novel therapeutic target for endometriosis-related ovarian neoplasms

Shahataj Begum Sonia[1], Kentaro Nakayama[2*], Sultana Razia[3], Naomi Nakayama[4], Masako Ishikawa[1], Hitomi Yamashita[1], Kosuke Kanno[1], Haruo Takeshita[3], Umme Farzana Zahan[1], Hasibul Islam Sohel[1], Satoru Kyo[1*]

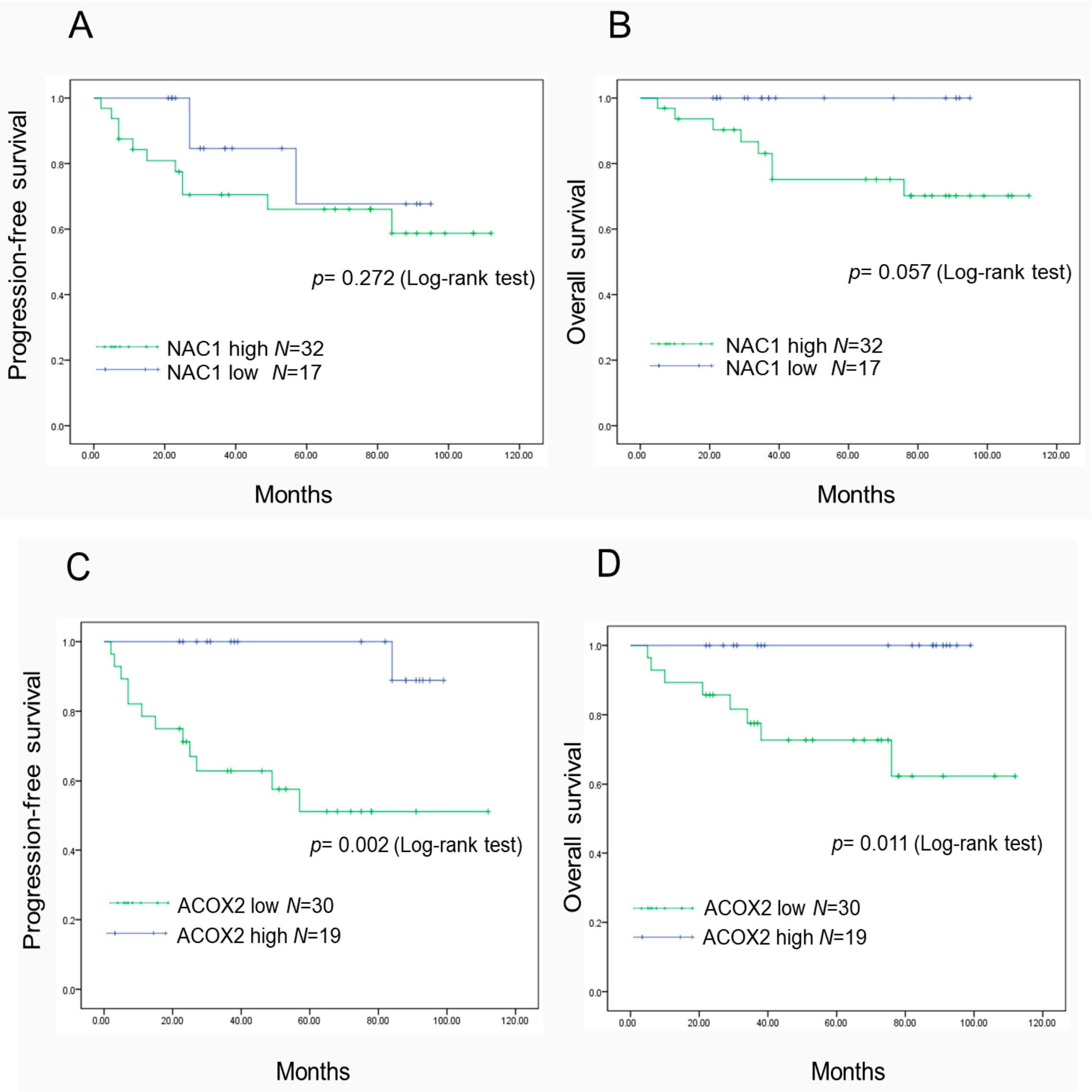

Supplementary Figure S1: Prognostic significance of NAC1 and ACOX2 expressions in patients with ERONs. Kaplan–Meier analysis of progression-free survival (PFS) (A) and overall survival (OS) (B) based on NAC1 expression. Kaplan–Meier analysis of progression-free survival (PFS) (C) and overall survival (OS) (D) based on ACOX2 expression. M: months.

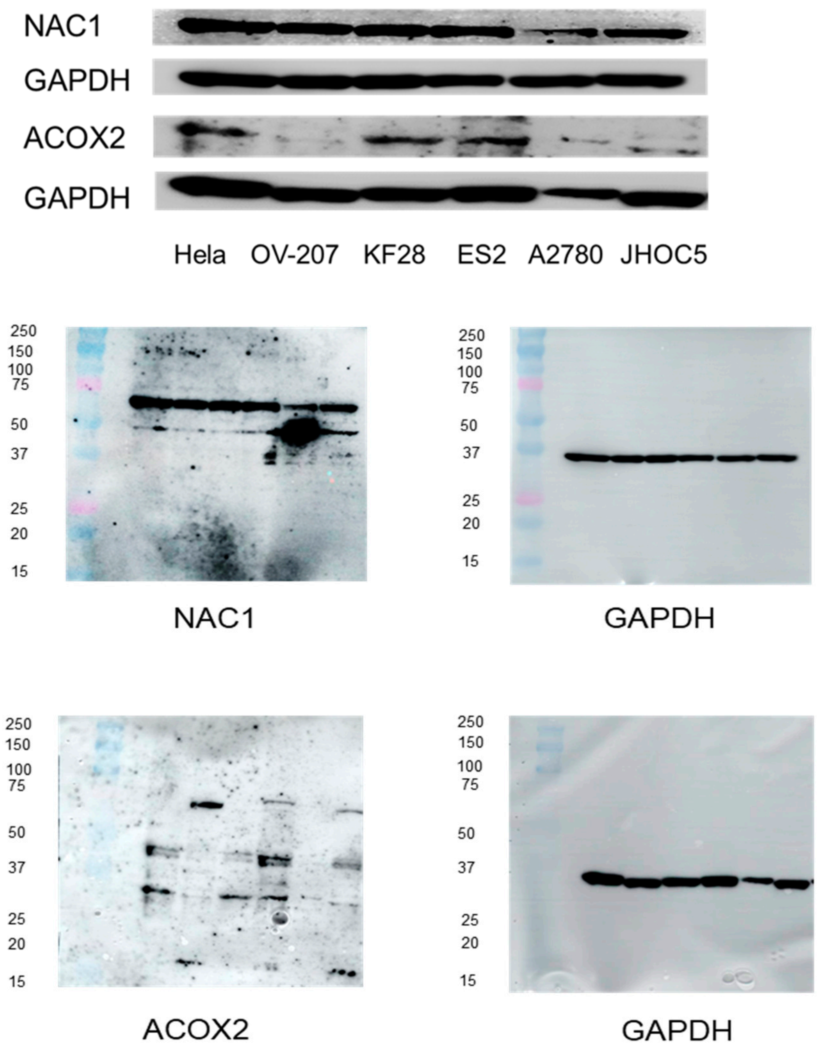

Supplementary Figure S2: Western blot images showing NAC1 and ACOX2 expression in OCCC cell lines. The molecular weights of NAC1, ACOX2 and GAPDH are 57kDa, 37.1kDa and 37kDa, respectively.

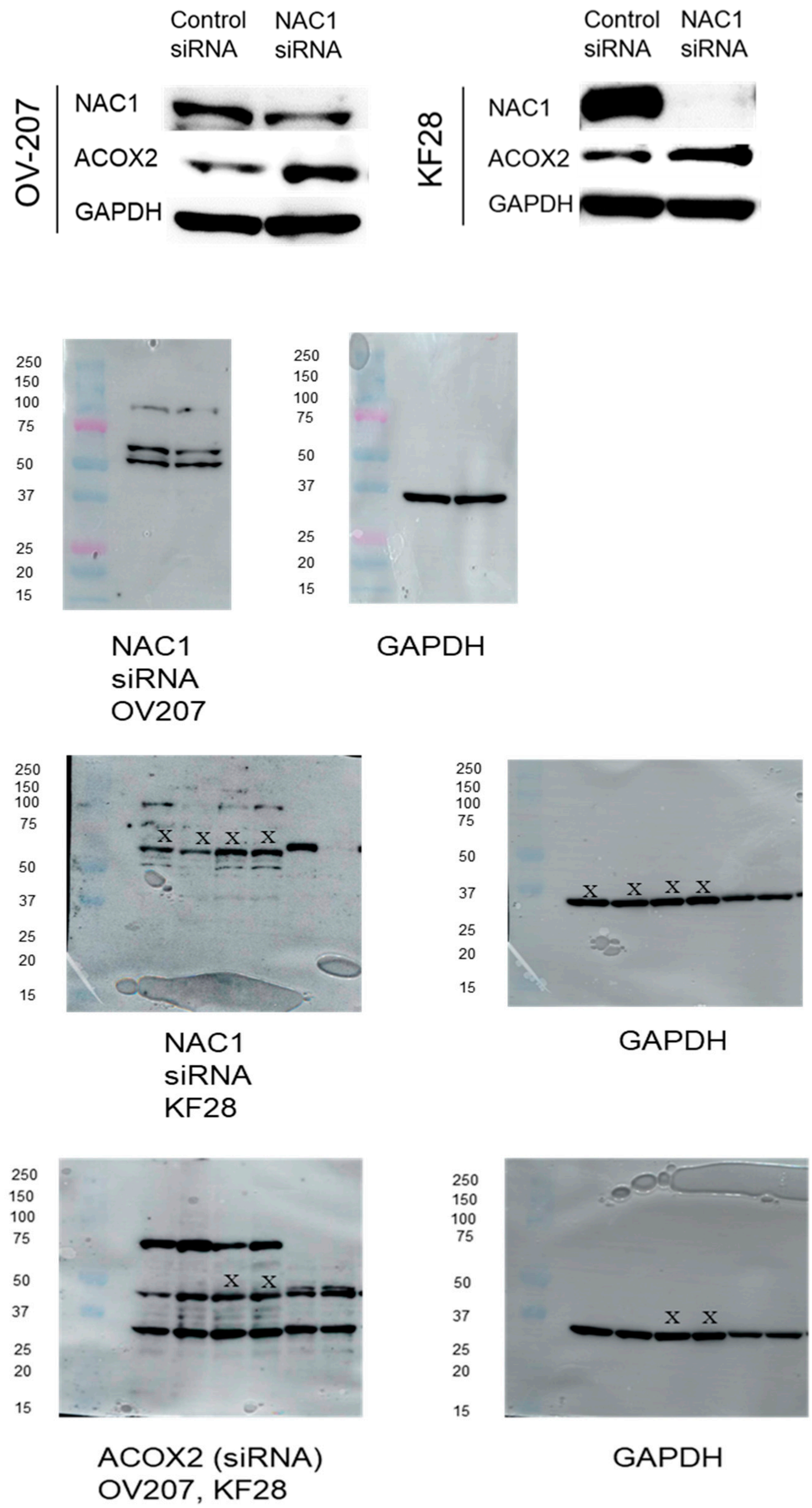

Supplementary Figure S3: Western blot analysis for NAC1 and ACOX2.

Expressions of NAC1 and ACOX2, of which molecular weight are 57kDa and 37.1kDa, respectively, are shown in OV207 and KF28 OCCC cell lines after siRNA knockdown.
